# Integrating Image Analysis and Machine Learning for Moisture Prediction and Appearance Quality Evaluation: A Case Study of Kiwifruit Drying Pretreatment

**DOI:** 10.3390/foods13121789

**Published:** 2024-06-07

**Authors:** Shuai Yu, Haoran Zheng, David I. Wilson, Wei Yu, Brent R. Young

**Affiliations:** 1 Department of Chemical & Materials Engineering, University of Auckland, Auckland 1010, New Zealand; syu981@aucklanduni.ac.nz (S.Y.);; 2 Electrical and Electronic Engineering Department, Auckland University of Technology, Auckland 1010, New Zealand

**Keywords:** kiwifruit drying, pretreatment, image processing, HSV color space, principal component analysis, partial least squares, random forest

## Abstract

The appearance of dried fruit clearly influences the consumer’s perception of the quality of the product but is a subtle and nuanced characteristic that is difficult to quantitatively measure, especially online. This paper describes a method that combines several simple strategies to assess a suitable surrogate for the elusive quality using imaging, combined with multivariate statistics and machine learning. With such a convenient tool, this study also shows how one can vary the pretreatments and drying conditions to optimize the resultant product quality. Specifically, an image batch processing method was developed to extract color (hue, saturation, and value) and morphological (area, perimeter, and compactness) features. The accuracy of this method was verified using data from a case study experiment on the pretreatment of hot-air-dried kiwifruit slices. Based on the extracted image features, partial least squares and random forest models were developed to satisfactorily predict the moisture ratio (MR) during drying process. The MR of kiwifruit slices during drying could be accurately predicted from changes in appearance without using any weighing device. This study also explored determining the optimal drying strategy based on appearance quality using principal component analysis. Optimal drying was achieved at 60 °C with 4 mm thick slices under ultrasonic pretreatment. For the 70 °C, 6 mm sample groups, citric acid showed decent performance.

## 1. Introduction

Over the past several years, the consumption of fruit has risen due to the increasing awareness of their nutritional properties and appreciable content of essential vitamins, minerals, and other nutrients beneficial to human health. As fresh fruit is highly perishable, extending its shelf life has emerged as a significant challenge [1]. Drying, or dehydration, is one of the oldest and most common processing technologies that, by reducing the moisture content, extends the shelf life by inhibiting microbial proliferation and diminishing the rate of deteriorative chemical reactions [2]. Furthermore, dried fruit has lower transportation and storage costs due to reductions in weight and volume [3,4,5].

Currently, hot air drying is the most applied method for drying processes in the food industry. Despite its lower drying efficiency compared to that of other new drying methods like freeze drying, the cost advantage is more important to producers. However, the deleterious effects of hot air drying on the physical, structural, chemical, organoleptic, and nutritional properties of foods can lead to a significant diminishment in consumer acceptability [2]. The shape, color, taste, odor, and nutritional composition of food products are all important indicators that help drive sales and profits [6]. Notably, when the appearance attributes of dried products deviate substantially from their fresh counterparts, they may fail to elicit the desired consumer appeal and purchasing intent. Consequently, preserving the external physical qualities is of paramount importance to render processed fruits attractive and acceptable to consumers. In practical production settings, most producers rely on subjective human visual evaluation or conventional measurement instruments to assess the appearance quality of the products. Although techniques employing instruments such as Lab* colorimeters or spectrophotometers provide accurate quantification of color, they are limited in scope as they assess only a partial area of the sample, rendering them impractical for the simultaneous evaluation of an entire batch of fruit [7]. The exigency of the continuous monitoring of appearance quality attributes in food processing necessitates the development and implementation of rapid, nondestructive testing methodologies.

Computer vision systems (CVSs) are considered efficient for quality control. Numerous investigations have documented the prospective applicability of CVSs in monitoring alterations in the visual characteristics of various dehydrated food products, including apples [8], ginseng [9], blueberries [10], bananas [11], and peas [12]. However, previous studies have primarily focused on using the CIELAB color space to evaluate color changes [13,14]. Few studies have attempted to use the HSV color space. Quevedo et al. [15] reported that CIELAB and HSV color spaces perform well regarding food color monitoring as they effectively represent the human eyes’ perception of color. Basak et al. [16] employed color features obtained from three color spaces (RGB, HSV, and HSL) in conjunction with a machine learning model to predict the total soluble solids and pH value of strawberry fruits. Their findings indicated that the HSV-based support vector machine regression (SVM-R) model exhibited the highest predictive accuracy. Pardede et al. [17] employed four color spaces, namely, RGB, HSV, HSL, and Lab*, in conjunction with a support vector machine (SVM) algorithm to assess fruit ripeness. Their results demonstrated that the HSV color feature exhibited the highest accuracy in determining the stage of fruit ripeness. Consequently, we investigated the potential of the HSV color space as a viable approach for monitoring and characterizing the color alterations occurring during food drying processes.

In addition to color characteristics, most previous studies also calculated shrinkage by comparing area retractions [10]. The shrinkage phenomenon occurring in foods during the drying process is irregular and anisotropic, rendering it challenging to accurately characterize the shrinkage behavior solely based on the changes in the projected area. The shape factors of 2D images can be considered more accurate quality indicators for describing irregular shrinkage. Only a few studies have focused on morphological changes during drying. Ding et al. [18] investigated the effect of morphological differences on the stacking density of milk powder. Nasri and Belhamri [19] considered shape factors’ influence on potato-case solar drying in drying kinetics. In general, the development and implementation of an image batch processing method, integrated into food drying equipment, holds significant potential for a wide range of industrial and research applications.

Kiwifruit (*Actinidia deliciosa* cv. Hayward) plays an essential role in New Zealand’s agricultural products and is exported to over 60 countries worldwide each year [20]. It is rich in nutritional elements, especially ascorbic acid, thus being attractive to consumers [13,21]. Improving the quality of the appearance of dried fruit can be achieved not only by optimizing the processing conditions but also by adding pretreatment steps. Some pretreatment methods for food before dehydration have been used, such as chemical methods (hyperosmotic, alkali, sulfite, and acid), gas treatments (sulfur dioxide, carbon dioxide, and ozone), thermal blanching (hot water, steam, super-heated steam, and ohmic and microwave heating), and nonthermal processes (ultrasound, freezing, pulsed electric field, and high hydrostatic pressure) [22,23,24]. However, sulfite pretreatment of food products is discouraged because its residues pose health risks when consumed [25]. Consequently, chemical pretreatment options, which have no documented health risks but are readily and widely available, such as citric acid and sodium chloride treatment, have been suggested [22]. In the food industry, hot water blanching is widely employed as a pretreatment [26]. Dandamrongrak et al. [27] also explored the effect of four pretreatments (blanching, freezing, chilling, as well as combined blanching and freezing) on the drying of bananas and found that the blanched sample was most preferred in terms of color. However, hot water blanching may have other adverse effects, such as on texture and flavor. In addition, ultrasonic pretreatment is a method being investigated as a likely alternative to conventional pretreatment. It has been shown to improve drying speed by altering plant tissue microstructure and product quality by reducing drying time [28,29]. With ultrasound, the internal structure of the food tissue is greatly altered, facilitating the transfer of water from the inside to the outside [30,31]. Pan et al. [32] confirmed that ascorbic acid and citric acid improved the color of banana crisps in freeze drying. Limited investigations have been conducted on the impact of nonthermal pretreatment methods on the drying process of kiwifruit. Nadian et al. [33] confirmed that acid ascorbic could prevent the browning of kiwifruit during the initial drying stage. Investigating the optimal pretreatment methodologies for drying kiwifruit presents an intriguing research avenue worthy of further exploration.

This study was segmented into three parts. Most notably, we developed an image batch processing method premised on the HSV color space, which facilitates the extraction of both color and morphological features. The accuracy of feature extraction was validated through a case study encompassing drying experiments on pretreated kiwifruit samples. In addition, we constructed moisture ratio prediction models by employing continuous image features extracted within a rolling time window (in half-hourly intervals) as input variables, utilizing partial least squares (PLS) regression and random forest (RF) regression. Ultimately, the optimal pretreatment method and drying conditions were determined by employing an unsupervised learning approach, principal component analysis (PCA), with the image features extracted at the end of the drying process serving as the input data. 

The main contributions of this study are as follows:

(1) We developed of an automatic appearance feature extraction method based on the HSV color space. This method exhibits substantial potential for integration into processing equipment, thereby enabling the real-time monitoring of food appearance during actual production processes in the future.
(2) We proposed an alternative approach with monitoring moisture loss by providing a means to assess image characteristics through more convenient analytical techniques.
(3) Based on the image feature data at the end of drying for each set of experimental samples, we used this measurement to quickly optimize the drying process by establishing good operating conditions.

## 2. Materials and Methods

### 2.1. Image Feature Extraction Batch Processing Steps

**Figure 1:**
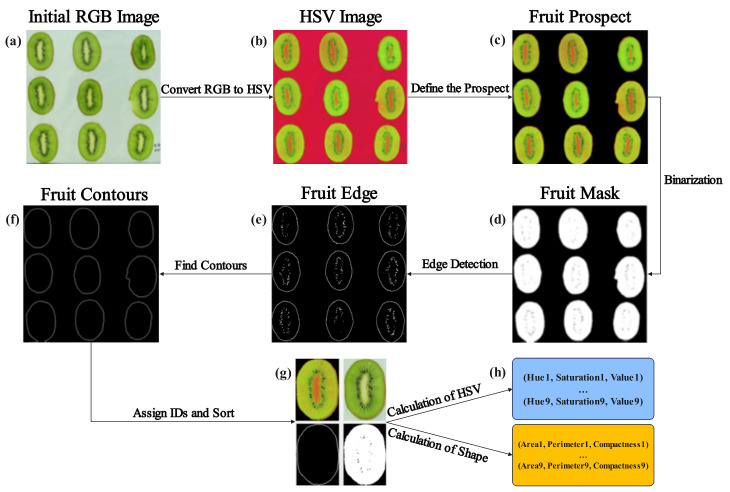
The steps of feature extraction from kiwifruit slice samples: (**a**) the original RGB image; (**b**) RGB image converted to HSV; (**c**) HSV-based background removal; (**d**) mask image of samples; (**e**) edge detection results; (**f**) contour extraction results; (**g**) sorted each slice according to their respective spatial interrelationships; (**h**) quantification of colorimetric and morphological attributes.

In this study, the images were processed using an algorithm written in the Python environment (version 3.8). To extract and quantify pertinent features from images for integration into machine learning models, we devised a feature extraction batch processing method. Figure 1 illustrates the complete workflow of our proposed image feature extraction steps.

In the dried kiwifruit pretreatment experiment, our aim was to obtain continuous image feature data during the drying process, including color and morphology. We chose the HSV color space to quantify the color change in kiwifruit. Subsequently, based on the significant color difference between the kiwifruit slices and the background, we segmented the prospect and background by setting a threshold range for the HSV color space. Subsequently, we used the ‘Mask’ function in the OpenCV library to create a mask, setting the pixel values within the specified color range to white and the rest to black. Edge detection was performed based on ‘cv2.Canny’ function. 

To quantify the evolution of their appearance characteristics throughout the drying process, it was imperative to establish a systematic correspondence between the same slices across different time points. This necessitated the assignment of unique identifiers (IDs) to each slice, thereby enabling the tracking of individual slices across a sequence of images. The underlying rationale for this approach was predicated on the observation that the relative positions of the kiwifruit slices remain largely invariant during the drying process. Consequently, we proposed the following methodological steps to facilitate the accurate matching and tracking of individual slices:

Step 1: Ascertain the coordinate values of the centroidal points for all bounded contour regions present within the image.

Step 2: Partition the bounded contour regions into three distinct subgroups based on their respective y-coordinate values.

Step 3: Sort each subgroup in ascending order according to the respective x-coordinate values of the constituent bounded contour regions.

Step 4: Assign unique identifiers (IDs) to each bounded contour regions.

Upon assigning the IDs, all images acquired during a given desiccation experiment were treated as a temporally ordered sequential dataset. The culminating step entailed the extraction of features for each individual kiwifruit section.

#### 2.1.1. Color Feature Extraction

A conical space model can be used to describe HSV color space. Hue (*H*) refers to the color or is equivalent to the wavelength of the transmission or reflection of the object. Traditionally, the value for hue is represented around a circle with a value spanning 0 to 360 degrees. Saturation (*S*) represents how close the color is to the spectral color and is approximately equivalent to spectral purity. The value range is 0–100%. The larger the value, the more saturated the color. Value (*V*) refers to the brightness of the color, usually in the range of 0–100% (black to white) [34]. In Python’s OpenCV library, *H* ranges from 0 to 180, and *S* and *V* range from 0 to 255. The thresholds for HSV were *H* (35–75), *S* (50–255), *V* (50–255), which were determined by iterative testing to ensure that kiwifruit contours could be accurately extracted throughout the drying process.

For RGB to HSV, the first step is scaling the RGB values to between 0 and 1. Then, the RGB image is converted to HSV with the following equation:

(1) $$H=\left\{\begin{array}{c} \;0,\;if\;Max=Min\\ \left(\frac{G-B}{Max-Min}\right)\times 60+0,\;if\;Max=R\\ \left(\frac{B-R}{Max-Min}\right)\times 60+120,\;if\;Max=G\\ \left(\frac{R-G}{Max-Min}\right)\times 60+240,\;if\;Max=B \end{array}\right.$$

(2) $$S=\left\{\begin{array}{c} 0,\;if\;Max=0\\ \left(\frac{Max-Min}{Max}\right),\;others \end{array}\right.$$

(3) V=Max (R,G,B)

where *Max* = max (R, G, B); *Min* = min (R, G, B).

#### 2.1.2. Morphology Feature Extraction

In this study, three shape factors (area, perimeter and compactness) were considered. Compactness is used to describe the shape characteristics during drying. When the fruit is perfectly round, its compactness reaches a maximum value of 1. As the shape of the fruit becomes more noncircular, the compactness tends to 0. The formulae for the three morphological features are as follows:

(4) $$Area=\frac{{Pixel\;of\;Area}_{t}}{Pixel\;of\;{Area}_{t0}}$$

where *Area_t_* and *Area_t0_* are the current fruit area fruit and fruit area before drying by counting the number of pixels;

(5) $$Perimeter=\frac{{Pixel\;of\;Perimeter}_{t}}{Pixel\;of\;{Perimeter}_{t0}}$$

where *Preimeter_t_* and *Preimeter_t_*_0_ are the current fruit perimeter fruit area before drying by counting the number of pixels.

(6) $$Compactness=\frac{{4\pi \times Area}_{t}}{{{Perimeter}_{t}}^{2}\;}$$

### 2.2. Drying Experiment of Case Study

#### 2.2.1. Sample Preparation

In the present study, kiwifruits (*Actinidia deliciosa* cv. Hayward) were purchased from a local supermarket in Auckland, New Zealand. When purchasing, samples were chosen that were similar in color and size, moderately hard, and had no obvious external defects. All the samples were stored in a refrigerator at 4.0 °C ± 0.5 °C to delay oxidation and deterioration caused by changes in characteristics. Before the drying experimentation, the samples were transferred from the refrigerator and kept for 3 h at room temperature. After that, fresh kiwifruits were washed to remove foreign matter and cut into the target thickness using a stainless-steel precision slicer, which could ensure a consistent thickness of the kiwifruit slices. To determine the initial moisture content of the kiwifruit slices, approximately 50 g of kiwi sample was placed in an oven and dried at 105 °C until a constant weight was achieved. This procedure was repeated three times, and the average value of the measurements was calculated. The initial moisture content of the sample in this study was 81% ± 0.07% on a wet basis.

#### 2.2.2. Experiment Design

The experiment employed a full factorial design, with two different desiccation temperatures (60 °C, 70 °C) and two distinct kiwifruit slice thicknesses (4 mm, 6 mm) as the treatment factors. Additionally, three pretreatment methods were implemented: immersion in a citric acid solution (CA), immersion in a sodium chloride solution (SCS), and ultrasonic pretreatment (ULT), along with a nonpretreated control group (Control). The specific experimental conditions are delineated in Table 1. To ensure the reliability and validity of the obtained results, each treatment combination was replicated in triplicate.

#### 2.2.3. Pretreatment Processes

(i) Citric Acid (CA)

For citric acid solution pretreatment, a 10 g/L solution of citric acid and water was used [31]. After weighing with a digital balance (Model: BL6001, Giorgio Bormac s.r.l, Carpi, Italy) and photographing, the sliced fruit samples were immersed in the solution for 10 min to ensure complete coverage. The fruit samples and solution had a weight ratio of 1 g/20 mL. The pretreated samples were taken out of the solution and blotted dry with a paper towel. After pretreatment, the samples were weighed and photographed again.

(ii) Sodium Chloride Solution (SCS)

The hypertonic solution pretreatment method used sodium chloride and water mixed with a ratio of 5 g/L to form the solution. The sliced fruit samples were soaked in the prepared solution at 0.1 g/mL for 30 min in a container to ensure complete coverage. The pretreated samples were then removed from the container and dried on a surface with a paper towel.

(iii) Ultrasound (ULT)

According to a previous study [35], the ultrasonic pretreatment method involved using an ultrasonic bath (Model: XUBA3, Grant instruments Ltd., Royston, United Kingdom) with a volume of 2.5 L and a fixed frequency of 44 KHz. The sliced fruit samples were immersed and treated with ultrasound in the machine for 30 min to ensure complete coverage. The surface was dried using a paper towel before sending to a tray dryer.

#### 2.2.4. Drying Process and Image Acquisition

The tray dryer used in this study was a UOP8 MKII (Armfield Ltd., Ringwood, UK). The drying air temperature could be easily controlled through a PID controller built into the dryer by a software (version UOP8 MKII Armsoft v.306), which varied the electrical supply to the heating element. The dryer was switched on and preheated for 30 min to reach the target temperature before drying, and the air velocity was set at 1.5 m/s. At the initiation of the drying process, the relative humidity of the hot air employed was approximately 22%.

As drying is an energy-consuming process, the time taken to reach the target moisture content is also a vital indicator of the suitability of the drying conditions. This experiment used five hours (300 min) as a reference time to monitor whether the samples had dried to a safe moisture level (moisture ratio = 0.05 ± 0.01) [33]. 

**Figure 2:**
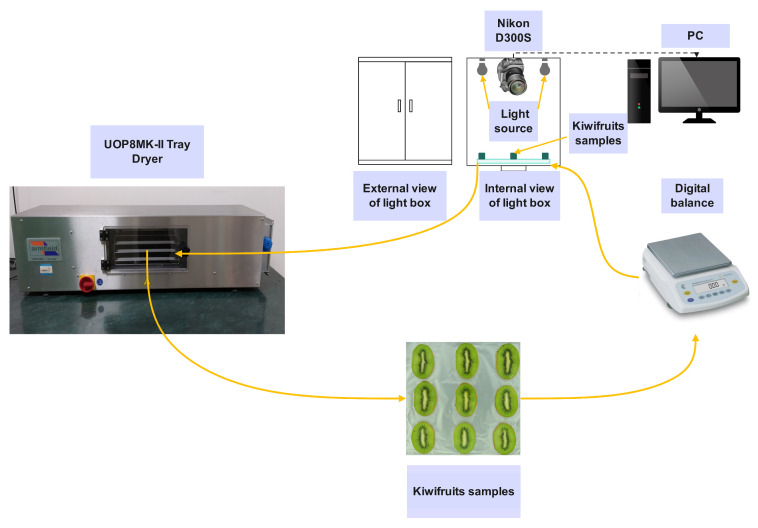
The diagram of experimental setup for image acquisition.

The image acquisition system consisted of a digital camera (Model: D300S, Nikon, Tokyo, Japan), lightbox with a 5500K planar LED array light source, and a personal computer (PC). The camera was securely mounted in a fixed position directly above the lightbox. During image capture, the sample specimens were precisely positioned beneath the camera’s lens for optimal imaging. A total of nine kiwifruit slices were placed on each tray in a 3 × 3 pattern, as shown in Figure 2. The camera was controlled by a PC connected to it, utilizing Camera Control Pro 2 software (version 2.37.1) for camera management and image acquisition. To ensure optimal image exposure, the camera settings were adjusted to an aperture value of f/5.6, an exposure time of 1/125 s, and an ISO sensitivity of 200. The acquired image had a resolution of 4288 × 2848 pixels. Kiwifruit sample weights were recorded half-hourly for the calculation of moisture ratio, and photographs were taken simultaneously for further image analysis. After recording, the samples were quickly returned to the drying chamber to continue drying. The data acquisition process for the entire drying experiment is depicted in Figure 2. 

#### 2.2.5. Calculation of Moisture Ratio

The moisture content (*M_t_*) (g water g^−1^ dry matter) of kiwifruit slices was calculated as

(7) $$M_{t}=\frac{W_{t}-W_{d}}{W_{d}}$$

where $W_{t\;}$ and $W_{d}$ in grams (*g*) are the current weight and the dried weight of sample, respectively. The moisture ratio (*MR*) was calculated as

(8) $$MR=\frac{M_{t}-M_{e}}{M_{0}-M_{e}}$$

where $M_{t}$ and $M_{0}\;$ are the equilibrium moisture content and the initial moisture content, respectively. However, $M_{e}$ can be neglected for products having a high initial moisture content. Therefore, Equation (8) was simplified as follows:

(9) $$MR∼\frac{M_{t}}{M_{0}}$$

### 2.3. Image-Feature-Based Moisture Ratio Prediction

#### 2.3.1. The Case Study Dataset for Regression

Integrating the color and morphological characteristics with the parameters derived from the drying experiments facilitated the prediction of the moisture ratio of the fruit slices, as delineated in Table 2. 

#### 2.3.2. Partial Least Squares (PLS) Regression

Partial least squares (PLS) is often used as the primary regression technique for multivariate data to express the relationship between independent and dependent variables [36]. PLS regression also has been used in the food industry. For example, Sahin and Demir [37] applied PLS to the determination of the antioxidant capacities of fruit juices. Sun et al. [38] developed a flavor prediction model for garlic using PLS.

In this study, PLS regression models were developed to predict the moisture ratio of kiwifruit samples during the drying process by using color and morphological features. K-fold cross-validation is a model validation technique that assesses the generalizability of a machine learning model to an independent dataset [39]. In the context of this study, the dataset was divided into *k* = 5 folds, and model performance was assessed using established evaluation metrics, with each fold alternately serving as the test set.

The model evaluation metrics for regression tasks include the multiple correlation coefficient (*R*^2^), the cross-validated R-squared (*Q^2^*), and the Mean Squared Error (*MSE*), which can be calculated by Equations (10)–(12), respectively:

(10) $$R^{2}=1-\frac{{SS}_{res}}{{SS}_{tot}}$$

where *SS_res_* is the residual sum of squares; *SS_tot_* is the total sum of squares. The closer *R*^2^ is to 1, the better the model fits the data.

(11) $$Q^{2}=1-\frac{PRESS}{{SS}_{tot}}$$

where PRESS means predictive residual sum of squares;

(12) MSE=1n∑i=1n(yi−y^i)2

where $y_{i}$ is the actual value, y^i is the predicted value, and n is the number of samples.

#### 2.3.3. Random Forest (RF) Regression

Random forest is an ensemble technique capable of performing both regression and classification tasks using multiple decision trees and a method called bootstrap aggregating [40]. The fundamental idea is to combine multiple decision trees to determine the final output rather than relying on a single decision tree. Random forests generate a large number of classification trees. New objects are classified by inputting vectors into each tree in the forest. Each tree assigns a classification, a process known as “voting”, and the classification with the highest number of votes is selected. 

In this study, random forest regression was used to make moisture ratio predictions for each kiwifruit slice during the drying process. We exploited the facilities of the scikit-learn library in the Python environment to train a random forest model comprising 20 decision trees. The model evaluation metrics for regression tasks includes mean absolute error (*MAE*), root mean squared error (*RMSE*), and multiple correlation coefficient (*R*^2^). The equation for *R^2^* is the same as above, and the other two are calculated as follows:

(13) RMSE=1n∑i=1n(yi−y^i)2

where $y_{i}$ is the actual value, y^i is the predicted value, and n is the number of samples.

(14) MAE=1n∑i=1nyi−y^i

Feature importance refers to the relative significance of each input variable in determining the prediction of a machine learning model. This concept is crucial for model interpretation, as it helps identify the most critical variable. 

The dataset and cross-validation methods used for training the random forest algorithm are consistent with those in Section 2.3.2.

### 2.4. Optimal Drying Strategy Determination by Principal Component Analysis

Principal component analysis (PCA) can compress high-dimensional index data into a low-dimensional space while retaining most of the critical information [41]. For drying processing, the new low-dimensional principal component space allowed for better observation of the differences and clustering among samples, which could guide the selection of optimal drying conditions.

In this study, the results obtained by using true values in the PCA were inaccurate due to the initial variations in the color and shape factors of the fruit slice samples. To minimize the impact of initial variation, all data were analyzed using the relative change before and after drying. The appearance features of the fresh fruits were determined as a reference group. After standardizing the color and morphological features data, PCA was employed to obtain the similarities in dried fruits under different drying conditions and pretreatment methods to determine the optimal drying strategy.

## 3. Results and Discussion

### 3.1. Visualization and Accuracy of Feature Extraction Batch Processing Method

**Figure 3:**
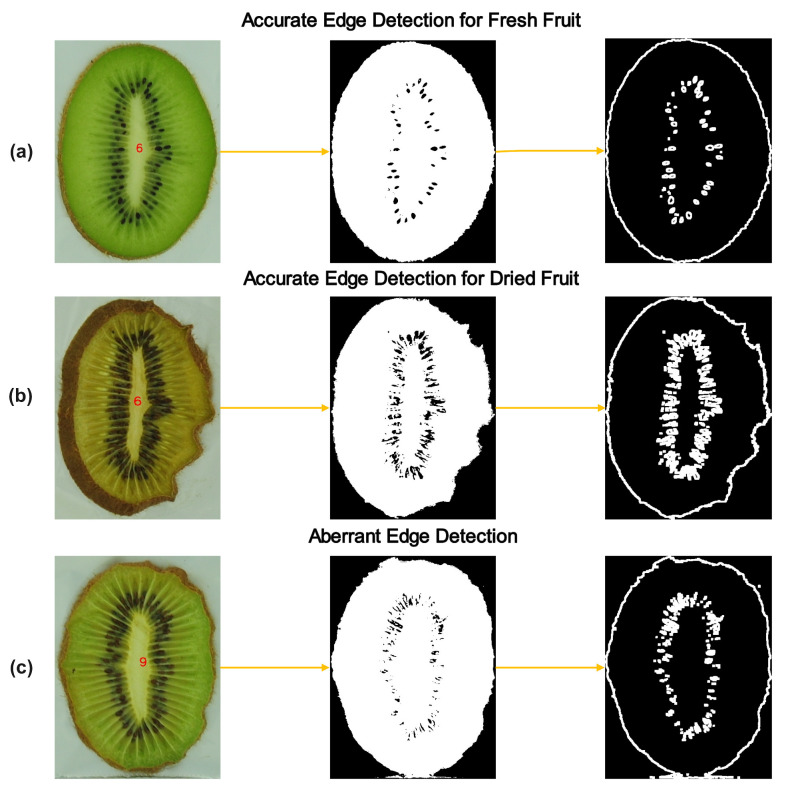
The diagrams of image edge detection for some samples: (**a**) accurate edge detection for fresh sample; (**b**) accurate edge detection for dried sample; (**c**) an example of an abnormal result.

Accurate image segmentation and edge detection are crucial for reliable image feature extraction, as inaccuracies can lead to inconsistent and erroneous morphological data. As illustrated in Figure 3a,b, our method accurately identified the correct contours at all stages of kiwifruit drying in most instances. The method accurately extracted the contours between the fruit flesh and the background. The edges are clear and coherent, effectively delineating the shape features of the kiwifruit slices. 

However, there were exceptions where the image processing encountered problems with contour extraction accuracy, resulting in unsuccessful data ID matching and some instances exhibiting areas outside the expected boundary range. As depicted in Figure 3c, the lower edge of the kiwifruit slice was not segmented correctly, and a portion of the background was incorrectly incorporated as part of the slice. The main reason for this issue was that the color of the bottom part of the kiwifruit slice was very similar to the color of the tray, lacking a significant color difference.

To address the problem that appears in Figure 3c, improvements can be considered from the following aspects. Firstly, using a tray with a greater color difference or increasing the distance between the kiwifruit slice and the tray can be explored. In the future, we can also consider introducing more intelligent segmentation algorithms, such as methods that combine region growing, texture analysis, or deep-learning-based segmentation algorithms. By comprehensively utilizing various information such as color, texture, and spatial relationships, these algorithms can improve the segmentation performance in low-contrast regions.

After the image processing phase, a total of 2700 groups of features were identified. Following the exclusion of these abnormal data, a subset of 2459 groups of valid features was retained for the training of the machine learning model. 

The accuracy of the proposed method exceeds 90%, indicating its robust performance. While this method has the potential for application in various food processing contexts, the HSV threshold would need to be adjusted to accommodate specific conditions. However, this conclusion warrants further verification through comprehensive testing. In future applications, the method could be integrated into existing drying equipment to facilitate online dynamic drying appearance prediction and quality control.

### 3.2. Prediction of the Moisture Ratio during the Drying Process

#### 3.2.1. Partial Least Squares Model

A partial least squares (PLS) model was developed to predict the change in moisture ratio (MR) of kiwifruit samples throughout the drying process, utilizing appearance features and drying conditions as variables. 

**Figure 4:**
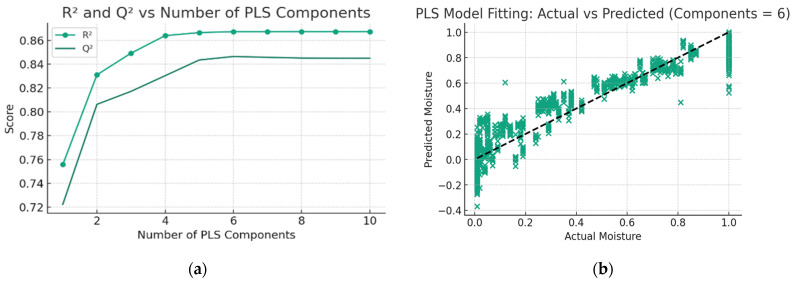
(**a**) *R*^2^ and *Q*^2^ versus the number of PLS components for the PLS model; (**b**) predicted MR versus actual MR during drying for kiwifruit samples.

Figure 4a delineates the variation in *R*^2^ and *Q*^2^ values concomitant with the increase in the number of PLS components. The *R*^2^ value is indicative of a model’s fitting accuracy on the training dataset, whereas the *Q*^2^ value elucidates a model’s capacity for generalization, as inferred from cross-validation procedures. Notably, as the count of PLS components escalates to five, the *Q*^2^ value culminates at a peak of 0.85, concurrently with the *R*^2^ value reaching 0.87. Complementarily, Figure 4b presents a comparative analysis of the predicted versus actual MR for all the dried kiwifruit samples. The model exhibits a mean squared error (*MSE*) of 0.017. These findings substantiate the efficacy of the PLS model in accurately predicting the moisture ratio of kiwifruit during the drying process.

#### 3.2.2. Random Forest Model

**Figure 5:**
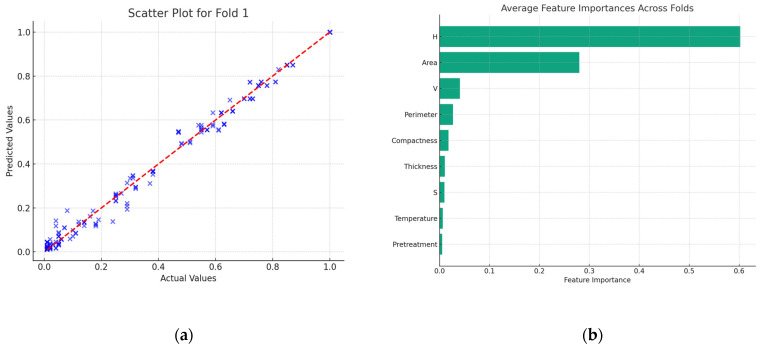
(**a**) Predicted MR versus actual MR during drying for fold one; (**b**) feature importance of random forest regression.

In this study, the monitoring of weight alterations and the image capture of kiwifruit samples were undertaken on a half-hourly basis. A random forest regression model was developed, aiming to predict the moisture ratio utilizing HSV color and morphological features. The model’s accuracy is depicted in Figure 5a; the disparity between the model’s predictions and the actual values is remarkably minimal. 

Further, a detailed analysis of the significance of each feature within the model was executed, as delineated in Figure 5b. This analysis highlighted ‘Hue’ as the most influential factor, with its importance score exceeding 0.6. Following closely was ‘Area’, with a significance score around 0.3. Other attributes such as ‘Value’, ‘Perimeter’, and ‘Compactness’ also emerged as factors of substantial importance. These empirical results are in harmony with our initial hypotheses. A strong correlation was observed between the color shift and area reduction in kiwifruit slices during the drying process with the variations in the moisture ratio. 

The model evaluation metrics in Table 3 show that the model demonstrates exceptional performance across all folds. The mean *R*^2^ of this model is very close to 1 (0.9923), indicating that the model explains a substantial proportion of the variance in the data. Both the *MAE* (0.0211) and *RMSE* (0.0312) are very small. 

This suggests that the image features for predicting the moisture ratio during the drying process in kiwifruit slices are highly effective.

To summarize, both the PLS and RF models presented above were relatively accurate in predicting the moisture ratio of kiwifruit samples during drying. Meanwhile, the prediction results of the models demonstrate the applicability of using color and morphological features to predict the moisture ratio of kiwifruit slices in real time. In the food industry, the confirmation of the drying endpoint is mainly performed manually based on experience. The use of these two types of models in this study may help with constructing soft sensors in the future. There is the potential to predict whether drying is complete by monitoring appearance changes during the drying process.

### 3.3. Effect of Different Pretreatments on Appearance (Color and Morphology) Changes

The moisture ratio (MR) of all samples was calculated using Equation (9), and it was found that all 60 °C, 6 mm samples did not reach the target MR after 300 min of drying. As mentioned above, a drying time of 300 min was used as a reference to represent the maximum acceptable drying time, with an MR equal to 0.05 representing the end of drying in this study. Therefore, the appearance change in the samples (60 °C, 6 mm) is not discussed as the drying was not completed. Section 3.3.1 discusses the similarity of the appearance of the 4 mm slice thickness samples at different drying temperatures (60 °C and 70 °C) to the fresh kiwifruit slices. Section 3.3.2 discusses the similarity of the appearance of samples with different slice thicknesses (4 mm and 6 mm) compared to fresh kiwifruit slices after drying at 70 °C. The PCA data were normalized. Thus, the coordinate system in the PCA figure and the Euclidean distance are dimensionless.

#### 3.3.1. Appearance Similarity Comparison by PCA for 4 mm Samples

**Figure 6:**
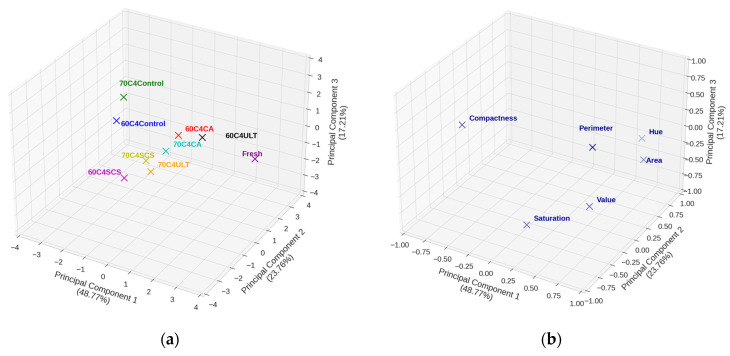
(**a**) PCA score plot of the first three principal components was used to explore the degree of variation in the appearance for different pretreatments for 4 mm samples; (**b**) PCA loading plot of the first three principal components allowing the determination of the relationships between different appearance variables.

The PCA scores and loadings plots for the 4 mm kiwifruits samples are shown in Figure 6a,b, respectively. The variance explained by PC1, PC2, and PC3 was 48.77%, 23.76%, and 17.21%, respectively, cumulatively accounting for 89.74% of the total variance. As shown in Figure 6b, regarding the appearance parameters, PC1 was positively correlated with hue, area, and value, while only negatively correlated with compactness. Figure 6a depicts a pronounced divergence of all dried sample groups from the fresh fruit (labeled as ‘Fresh’), signifying substantial alterations in appearance, attributable to the drying process. 

Table 4 presents the Euclidean distances between each sample point and the fresh fruit reference point. Notably, the points labeled 60C4ULT and 60C4CA are positioned proximate to those of the fresh fruit, suggesting that the application of citric acid and ultrasonic pretreatments at a drying temperature of 60 °C and a slice thickness of 4 mm potentially preserves green color effectively. In addition, the 70C4SCS and 70C4ULT groups are located close to each other in Figure 6a and moderately deviate from the fresh fruit point. This means they had similar changes in appearance at the end of drying. 

The control samples, irrespective of being dried at 60 °C or 70 °C, are distinctly departed from the fresh fruit in Figure 6a, indicating that regardless of the pretreatment used, it is beneficial to inhibit color and morphological changes during the process of drying kiwifruit.

**Figure 7:**
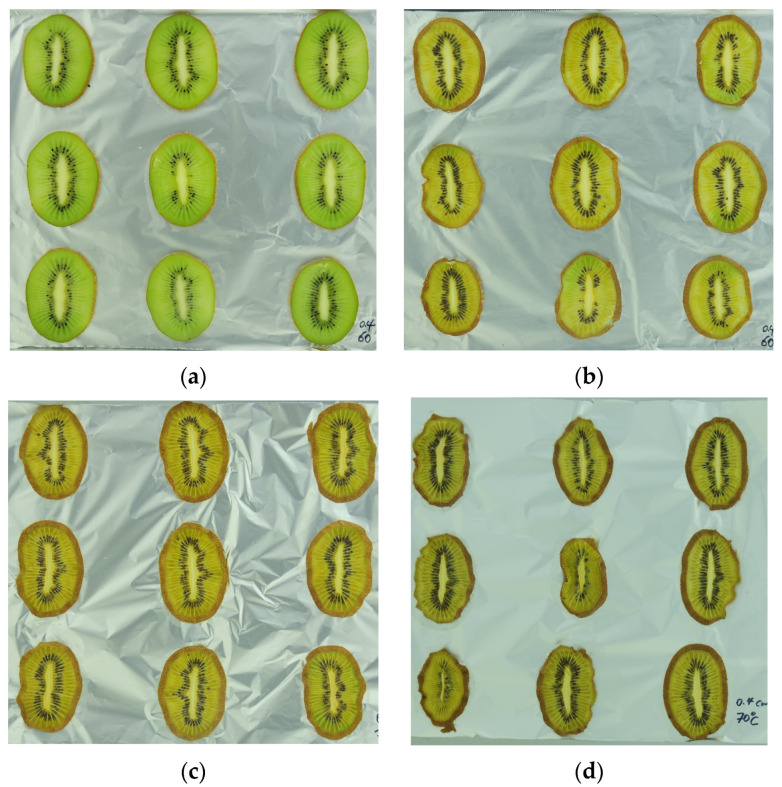
(**a**) Fresh fruit before drying; (**b**) 60C4ULT after drying; (**c**) 70C4CA after drying; (**d**) 70C4Control after drying.

A comparison of ultrasonic, citric acid, and unpretreated samples with fresh fruit is shown in Figure 7. The results of human eye perception are consistent with the data, showing that ultrasonic-pretreated samples best retained the green and had less morphological modification. In contrast, nonpretreated samples browned heavily after drying and showed a large degree of shrinkage.

In general, an analysis integrating the data from Figure 6 and Table 4 suggested that for the 4 mm sliced kiwifruit samples, ultrasonic pretreatment was optimal at a drying temperature of 60 °C. In contrast, citric acid pretreatment yielded favorable results at an elevated temperature of 70 °C. Furthermore, all selected pretreatment methods in this study mitigated appearance-quality deterioration to varying extents compared to the samples without pretreatment.

#### 3.3.2. Appearance Similarity Comparison by PCA for 70 °C Samples

**Figure 8:**
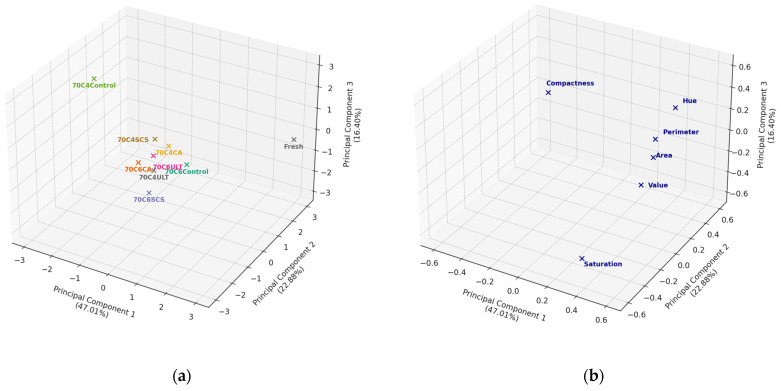
(**a**) PCA score plot of the first three principal components was used to explore the degree of variation in the appearance for different pretreatments for 70 °C samples; (**b**) PCA loading plot of the first three principal components allowing the determination of the relationships between different appearance variables.

Figure 8a,b show the PCA scores and loading plots for the 70 °C drying temperature of kiwifruit samples, respectively. PC1, PC2, and PC3 accounted for 47.01%, 22.88%, and 16.40% of the variance, cumulatively constituting 86.29%. Figure 8b shows that PC1 was positively correlated with all parameters except compactness, while PC2 had positive correlations with all parameters except saturation. 

It was observed that samples with the same pretreatment differed in appearance at the 70 °C drying temperature for different slice thicknesses. This discrepancy is likely attributable to the extended drying duration necessary for the 6 mm samples. This protracted drying period presumably intensified the degree of browning and shrinkage in the fruits, thereby engendering appearance differences among the sliced samples. As drying progresses, the moisture content of the fruit gradually decreases, leading to an acceleration of the Maillard reaction. As the Maillard reaction accelerates, interactions between sugars and amino acids at higher temperatures lead to the formation of more brown compounds [4]. However, the score plot shows the position of the individual samples in the principal component space, and, as can be seen in Figure 8a, most of the sample groups are relatively clustered in position between each other, which means that there was not much difference in the appearance features between the samples, except for the 70C4Control sample.

As in the previous section, the Euclidean distances of each sample from the fresh fruit were computed, as delineated in Table 5. The 70C4Control group exhibited the most pronounced appearance deviation from the fresh fruit, possibly due to the rapid drying of thinner slices at high temperatures, which precipitated significant pigment deterioration and shrinkage. The 70C4CA samples demonstrated the shortest Euclidean distance (3.84) from the reference point (Fresh). Remarkably, the unpretreated 6 mm samples (70C6Control) subjected to drying at 70 °C did not undergo severe appearance deterioration. This may have been because thicker samples are more likely to produce a porous hard outer case at higher temperatures, thereby retarding shape change [33].

**Figure 9:**
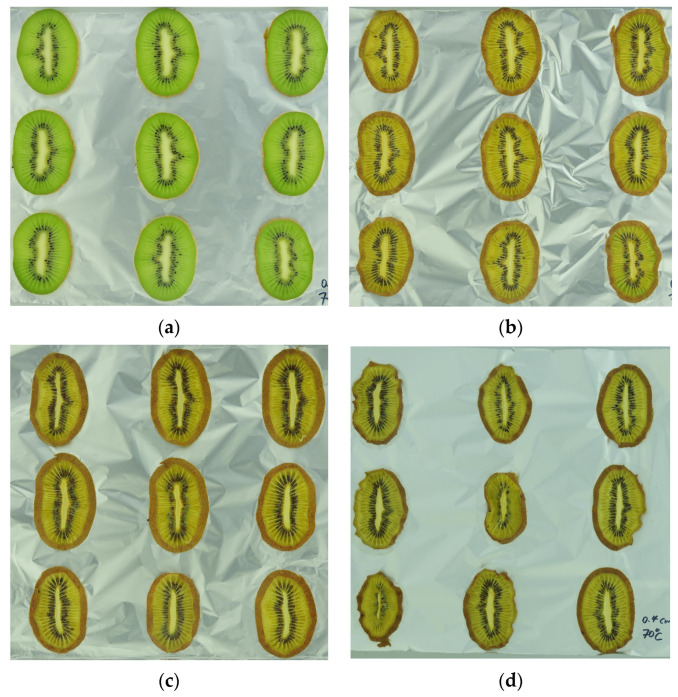
(**a**) Fresh fruit before drying; (**b**) 70C4CA after drying; (**c**) 70C6Control after drying; (**d**) 70C4Control after drying.

As shown in Figure 9, when the drying temperature was raised to 70 °C, the human eye perceived that only the citric-acid-pretreated samples (b) retained a little green color and did not undergo severe morphology changes. 

In summary, at a drying temperature of 70 °C, citric acid appeared to be the most efficacious pretreatment for the 4 mm slices. However, for thicker slices, the various pretreatments did not exhibit notably effective performance.

## 4. Conclusions

In this study, an image batch processing method was developed, facilitating the extraction of color and morphological features with an accuracy exceeding 90% for kiwifruit slices. This result indicates the potential to integrate such method into smart drying equipment and help optimize the drying process.

Furthermore, partial least squares (PLS) and random forest (RF) models were developed using color and morphological features to predict the moisture ratio. While the RF model exhibited superior predictive capability, PLS models, due to their greater explainability, might be more suitable for real-world industrial applications. The satisfactory prediction outcomes suggest vision-based soft sensors have potential application in monitoring moisture content in the food industry without relying on weighing equipment.

Principal component analysis (PCA) was employed to compare the color and morphological differences among the samples. Specifically, for kiwifruit slices with a 4 mm thickness that underwent ultrasonic pretreatment, minimal appearance changes were observed at 60 °C. However, when the drying temperature increased to 70 °C, the slices pretreated with citric acid closely resembled fresh fruit.

Overall, through the integration of image processing and machine learning techniques, the proposed approach presents an efficient and accurate method for monitoring the fruit drying process. However, this study is a case study of kiwifruit slice drying, and the general applicability of the real-time image feature extraction method to different types of food will be verified in future research. The application of these methodologies in dynamic fruit drying systems is anticipated to enhance the productivity and effectiveness of fruit drying operations.

## Figures and Tables

**Table 1:** Full factorial experimental design for kiwifruit.

Group Name	Drying Temperature (°C)	Slice Thickness (mm)	Pretreatment
60C4Control	60	4	None
70C4Control	70	4	
60C6Control	60	6	
70C6Control	70	6	
60C4CA	60	4	Citric acid solution
70C4CA	70	4	
60C6CA	60	6	
70C6CA	70	6	
60C4SCS	60	4	Sodium chloride solution
70C4SCS	70	4	
60C6SCS	60	6	
70C6SCS	70	6	
60C4ULT	60	4	Ultrasound
70C4ULT	70	4	
60C6ULT	60	6	
70C6ULT	70	6	

**Table 2:** The dataset structure.

Variable	Name	Range of Values
X_1_	Hue	0 to 180
X_2_	Saturation	0 to 255
X_3_	Value	0 to 255
X_4_	Area	0 to 1
X_5_	Perimeter	0 to 1
X_6_	Compactness	0 to 1
Y	Moisture Ratio	0 to 1

**Table 3:** Model evaluation by random forest regression.

Fold	*R^2^*	*MAE*	*RMSE*
1	0.9939	0.0185	0.0288
2	0.9920	0.0214	0.0317
3	0.9930	0.0205	0.0303
4	0.9920	0.0204	0.0307
5	0.9907	0.0242	0.0345
Mean	0.9923	0.0211	0.0312
Standard deviation	0.0011	0.0018	0.0019

**Table 4:** Euclidean distance to fresh fruit for 4 mm thick samples.

Number	Name	Distance to Fresh
1	60C4ULT	3.44
2	70C4CA	3.81
3	70C4SCS	4.46
4	70C4ULT	4.68
5	60C4CA	5.32
6	60C4SCS	5.51
7	60C4Control	5.54
8	70C4Control	5.66

**Table 5:** Euclidean distance to fresh fruit for 70 °C samples.

Number	Name	Distance to Fresh
1	70C4CA	3.84
2	70C6Control	4.41
3	70C6ULT	4.47
4	70C4ULT	4.51
5	70C4SCS	4.52
6	70C6SCS	4.85
7	70C6CA	4.89
8	70C4Control	6.63

## Data Availability

The data presented in this study are available on request from the corresponding author due to the data are part of an ongoing study.
